# Applications of Antioxidants in Dental Procedures

**DOI:** 10.3390/antiox11122492

**Published:** 2022-12-18

**Authors:** Fan Qi, Haofei Huang, Ming Wang, Weifeng Rong, Jing Wang

**Affiliations:** School of Chemistry and Chemical Engineering, Shandong University of Technology, 266 Xincun Road, Zibo 255000, China

**Keywords:** antioxidants, antioxidant delivery, dental procedures, tooth bleaching, dental implants, dental restorations

## Abstract

As people are paying more and more attention to dental health, various dental treatment procedures have emerged, such as tooth bleaching, dental implants, and dental restorations. However, a large number of free radicals are typically produced during the dental procedures. When the imbalance in distribution of reactive oxygen species (ROS) is induced, oxidative stress coupled with oxidative damage occurs. Oral inflammations such as those in periodontitis and pulpitis are also unavoidable. Therefore, the applications of exogenous antioxidants in oral environment have been proposed. In this article, the origin of ROS during dental procedures, the types of antioxidants, and their working mechanisms are reviewed. Additionally, antioxidants delivery in the complicated dental procedures and their feasibility for clinical applications are also covered. Finally, the importance of safety assessment of these materials and future work to take the challenge in antioxidants development are proposed for perspective.

## 1. Introduction

Since the 20th century, the prevention and treatment of oral diseases have made great progress, and the occurrence of dental caries and oral inflammation have also dropped significantly [1]. It is reported that many oral problems are related to an imbalance of antioxidants and reactive oxygen species (ROS) in the body. In recent years, free radicals have been found to be related to the occurrence and development of dental diseases, and antioxidants have also been used in dental treatment [2].

Free radicals and ROS are the products of oxidative stress and have extremely oxidative properties. The main sources of free radicals in the oral environment are considered as the following: food (high fat, high calorie), alcohol and cigarettes, dental treatment (surgery, laser, ultraviolet, etc.), dental materials (adhesive, composite resin, etc.), and periodontal diseases [3]. The antioxidant capacity in the oral environment of each person is different. Oxidative stress occurs when the body’s oxidative and antioxidant capacity is imbalanced and favors oxidation, which is also the main cause of oral and dental diseases. When reacting with antioxidants, free radicals will gain an electron and are converted into normal molecules [4], thereby reducing damage to the body.

Antioxidants prevent free radicals from requesting electrons from normal cells, and actively donate electrons to free radicals, thereby achieving the purpose of protecting normal cells. Antioxidants can also inactivate free radicals before they attack the body’s cells, and they play a supporting role in the treatment of oral problems such as periodontitis [5]. In addition, some researchers found that the intake of antioxidants can effectively inhibit the growth and reproduction of oral cancer cells [6,7]. Antioxidants are typically divided into two types: endogenous and exogenous. Endogenous means that they can be produced by the human body, including superoxide dismutase (SOD), catalase (CAT), and reduced glutathione (GSH). Exogenous means that the human body cannot synthesize them. Commonly used exogeneous antioxidants are ascorbic acid (vitamin C), tocopherol (vitamin E), quercetin, tannic acid, and N-acetyl cysteine (NAC). The mechanisms of action and delivery form of some exogenous antioxidants are shown in Table 1. A variety of delivery methods, such as encapsulation and sol–gel technology, have emerged [8,9,10]. During the transition process, the antioxidant might decompose due to its instability, leading to a decreased effectiveness. Although an appropriate amount of ROS has a bactericidal effect, a large number of decomposed antioxidants can adversely affect the treatment of diseases [11]. From this point, the biocompatibilities of current antioxidants also need to be addressed.

This review focuses on the involvement of antioxidants in dental procedures, such as dental bleaching, implants, and dental fillings. Types of antioxidants used to treat oxidative stress induced by dental treatments and restorations are covered, and the mechanism of action and delivery of antioxidants are also discussed. From the developmental perspective, the advantages and disadvantages of current antioxidants are evaluated, and future challenges are also proposed.

## 2. Reactive Oxygen Species (ROS) and Oxidative Damage

### 2.1. ROS in Oral Environment

ROS refers to an extremely powerful oxidant that includes free radicals and nonradical molecules [20]. In general, free radicals must be ROS, but ROS are not necessarily free radicals [21]. The atomic orbitals of free radicals contain unpaired electrons, which are very unstable and highly reactive.

ROS of physiological concentrations have positive effects [22]. In the medical field, the active oxygen produced by photolysis, photocatalysis, and photodynamic therapy is targeted for cancer therapy. In the dental field, an appropriate amount of ROS is used for the treatment of periodontal diseases and antibacterial photodynamic therapy of root canal disinfection. The hydrogen peroxide in tea and coffee is an example, which has a preservative effect [23]. In addition, an appropriate amount of ROS is also used as a dental bactericide for dental fillings [24]. Moderate amounts of free radicals are usually involved in immune responses and metabolism in certain parts of the body [25].

However, when stimulated, excess ROS may cause oxidative stress and, thus, disease. The most damaging radicals in the body include hydroxyl radicals, superoxide anion radicals, hydrogen peroxide, oxygen singlet, hypochlorite, nitric oxide free radicals, and peroxynitrite free radicals [4]. For an ROS, under aerobic conditions, a part of oxygen is converted into superoxide anion radicals and hydroxyl radicals through electron transfer; another part of oxygen is converted into hydrogen peroxide through electron reduction and accepts a proton. These highly reactive free radicals mainly damage deoxyribonucleic acid (DNA), proteins, carbohydrates, and lipids in the nucleus and cell membranes [26].

**Figure 1:**
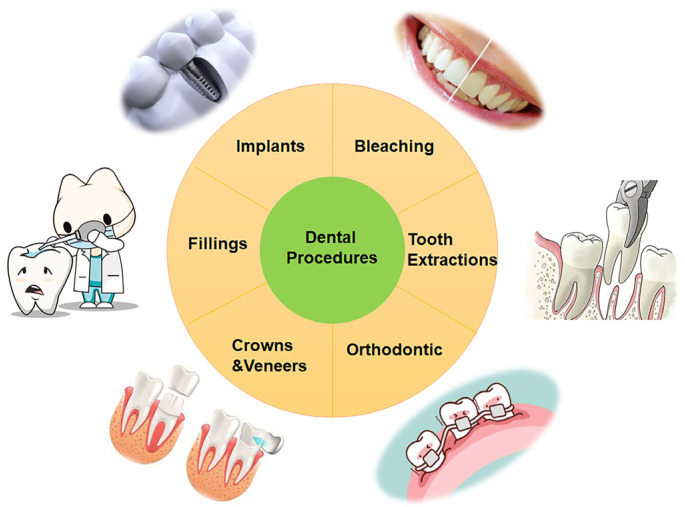
Sources of ROS production in dental procedures.

Sources of free radicals in oral environments include normal metabolic processes in the body and some external sources, including X-rays, ozone, smoking, and dental treatments. Oral inflammation is the main source of inflammatory response and the increase of ROS in oral tissues, gums, and saliva [25]. ROS are produced by phagocytic cells, such as mitochondria, to fight microbial invasion. In this process, a large amount of bactericidal superoxide anions can be produced, and the process of superoxide anion elimination produces hydrogen peroxide [20]. While causing local damage to the oral cavity, the free-radical-rich gingival fluid mixed with saliva stimulates the production of ROS in other oral tissues [27]. Oxidative stress in the oral cavity is mainly related to infection and inflammation of the gums and other soft tissues, but other factors may also lead to oxidative stress. The sources of ROS are also relatively wide, mainly including food, cigarettes, alcohol, dental materials, and drugs [3]. When drinking alcohol, the amount of ethanol, acetaldehyde, and ROS in the oral environment will increase rapidly, and after a period of time, acetaldehyde and ROS will remain at high concentrations [28]. Surprisingly, free radicals are produced at every stage of alcohol metabolism, including alcohol dehydrogenase and mitochondrial enzymes, which oxidize to superoxide anion radicals and hydroxyl radicals, respectively [29]. Whether it a chronic alcohol intake or one dose, it will affect the homeostasis of the oral cavity, reduce the activity of enzymes, and cause an increase in ROS [30,31]. There are thousands of toxic substances and hundreds of pro-oxidants in cigarette smoke [32]. According to the research, burning of a cigarette produces tar and smoke, 35 milliliters of cigarette smoke contains 10^15^ free radicals, and 1 g of tar has 10^17^ [33]. These free radicals may further promote the production of oxidants in saliva, change its proteins, and reduce the activity of antioxidant enzymes [34]. In addition, it has been reported that high-fat, high-calorie-fed rats have increased ROS production and reduced antioxidant barriers in the oral cavity [35]. The main source of ROS in the oral environment is periodontitis. At the same time, the oral cavity is also exposed to ROS produced by various dental materials, and some dental procedures, such as bleaching, implants, fillings, crowns, veneers, orthodontics, and tooth extractions, may also produce ROS (Figure 1).

As people are paying more attention to dental health, various dental treatment procedures are becoming popular, which also means that the sources of ROS are more extensive than expected.

### 2.2. Oxidative Stress and Oxidative Damage

Oxidative stress, the phenomenon of excess oxidants, is caused by an imbalance in the production of free radicals in the antioxidant system [36]. Theoretically, oxidative stress can be divided into basal, low-intensity, medium-intensity, and high-intensity oxidative stress according to the content of oxidants [21]. Short-term oxidative stress may occur in tissue loss such as trauma, infection, and heat damage. These damaged tissues produce increased activity of phagocytic free-radical-generating enzymes, release free metal ions, destroy epoxidation phosphorylated electron transport chain, and produce excessive ROS [4]. It has already been proven that oxidative damage of cellular components such as proteins, lipids, and nucleic acids may occur when ROS production increases or antioxidant capacity decreases [37]. Oxidative stress corresponds to many diseases, including cardiovascular disease, cancer, and various kinds of inflammation. Oxidation of the lipid component of low-density lipoproteins is an important factor in atherosclerosis [38]. At the same time, a large number of free radicals react with DNA, such as strand breaking base modification and DNA protein cross-linking, and induce DNA damage, resulting in cell mutation and cancer.

**Figure 2:**
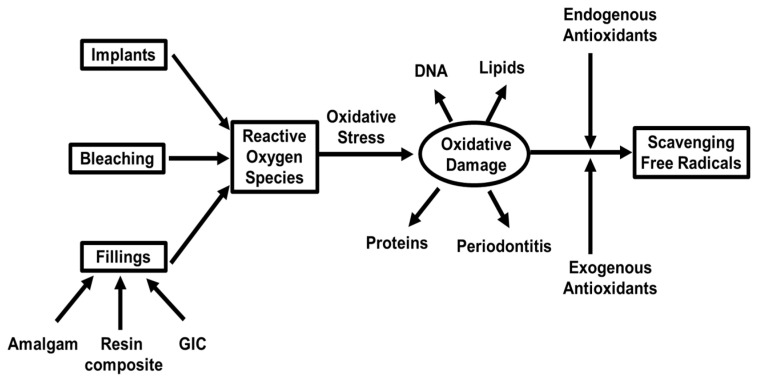
Schematic diagram of ROS production and clearance during dental procedures.

In the course of dental procedures, oxidative stress and oxidative damage also occur. It has been shown that periodontal inflammation is a direct result of increased ROS and oxidative damage products in the oral cavity [25]. Except for the factors of alcohol consumption and nicotine exposure, dental procedures such as implants, bleaching, and fillings also lead to oxidative damages (Figure 2). When exogenous coupled with endogenous antioxidants are utilized, free radicals are scavenged to reduce the oxidative damage. Periodontitis is a chronic inflammation of periodontal tissue, which can lead to the loss of alveolar bone or even tooth loss when it is serious, and oxidative stress is a part of the pathogenesis of periodontal disease [39,40]. The usual treatment is to scrape the tissues related to root gouging, which can successfully treat most periodontal diseases [41].

As for the oxidative stress phenomenon generated in dental procedures, antioxidants might be added to eliminate free radicals. Antioxidants are used to combat oxidative stress. Working mechanisms of antioxidants mainly include two types: one is the chain-breaking mechanism, that works through antioxidants to provide electrons to free radicals, so that free radicals become stable molecular structures without damage to normal cells; the other is to remove ROS or reactive nitrogen species (secondary antioxidants) by quenching chain-initiating catalyst [4]. Therefore, appropriate antioxidants must be chosen according to the species of free radicals. Among them, naturally extracted antioxidants are more popular.

## 3. Antioxidants Used in Dental Procedures

### 3.1. Tooth Bleaching

Tooth colors and structures might be affected by foreign pigments or drugs such as tobacco, tea, coffee, and so on. With the pursuit of aesthetics, tooth bleaching technology has attracted more and more attention. Tooth bleaching is the use of chemical principles, through the method of oxidative replacement by using oxidants, to replace the pigment in the tooth surface in order to achieve the purpose of tooth whitening. At present, the commonly used tooth bleaching agents are hydrogen peroxide and carbamide peroxide. In addition to a variety of bleaching substances, light sources such as allografts, lasers, light emitting diodes (LEDs), and ultraviolet lights can also be used to enhance bleaching [42].

Bleaching works by releasing ROS. The active ingredients of bleaching agents are 10–40% hydrogen peroxide or 10–22% carbamide peroxide [43], which whiten the tooth by using chemicals to oxidize organic pigments within the tooth structure. In the presence of metal ions such as iron ions, hydrogen peroxide can generate oxygen-derived free radicals, hydroxyl radicals, which have strong oxidation capacities [44]. In the process of tooth bleaching with hydrogen peroxide, hydroxyl radical plays the oxidative role. Hydroxyl radical does not affect the inorganic tissue of dentin, but does attack the organic components of dentin, thus achieving the purpose of whitening teeth [45]. As for carbamide peroxide, it is first decomposed into hydrogen peroxide in the mouth, and the bleaching mechanism is the same as described above. The heat generated by light sources is helpful to further activate the bleaching agents, and better bleaching effect is able to be obtained by better penetration of hydrogen peroxide into the tooth structure. However, deeper penetration of hydrogen peroxide may not only infiltrate the enamel and dentin, but also reach the dental pulp, which may lead to irreversible damage to tooth structures and pulp cells [46]. Soares et al. [47] demonstrated that pulp cells exposed to a certain concentration of bleaching gel were in a state of oxidative stress, the intensity of which was proportional to the contact time between the bleaching gel and enamel. Additionally, dental bleaching may cause tooth sensitivity and periodontal discomfort [48], and periodontal tissue damage may further cause root absorption, resulting in trauma or developmental defects in the cementum layer of the tooth neck [49]. If a nonvital tooth has been developed without response to electric pulp test, its discoloration can be managed by an internal bleaching. A clinical case report by Anugrahati et al. [50] demonstrated that the internal bleaching on a nonvital tooth was the best option to restore the function and aesthetics of the original tooth. At the same time, the hydroxyl radical is one type of oxygen-derived free radical and is considered to be extremely oxidizing, which can destroy connective tissue components, collagen, hyaluronic acid, etc. [51,52]. If hemoglobin is exposed to hydrogen peroxide, the iron in the hemoglobin molecule reacts with hydrogen peroxide, which produces hydroxyl radical (an oxygen-derived free radical), thereby destroying healthy hemoglobin [53]. Hydroxyl radicals are also able to alter DNA by strand breaks and damage cell membranes by lipid peroxidation [54]. When composite bonding is performed immediately after tooth bleaching, the bonding strength might be negatively affected [55]. The residue of peroxide in the bleaching procedure inhibits the polymerization and curing of adhesive resin materials [56]. The enamel pores and dentin can store peroxides, leading to a greatly increased concentration of peroxides on the enamel surface, which further prevents the complete curing of some resin materials and finally affects the effect of adhesive repair [57].

During tooth bleaching, oxidative stress occurs in the mouth due to the residual oxide caused by bleaching agents, causing damage to the human body, and also affecting the bonding to teeth after bleaching. The proper use of sealer base is the most direct approach to protect the periodontal area and limit the penetration to dental pulp. Even if sealer base has been applied, there are still risks that bleaching agents induce negative changes in tooth surfaces, such as reduction of microhardness, mineral loss, and surface roughness, where the residue oxygen radicals and peroxides after bleaching are considered as the main reason [58,59]. Antioxidants in saliva cannot remove these residual reactive oxygen species in a short time, and more than two weeks are needed to eliminate the effect [60]. Therefore, the use of antioxidants in the bleaching process is very important to neutralize the residual oxidants in the oral environment. Ascorbic acid is one of the most commonly used antioxidants, and cannot be synthesized by humans. Ascorbic acid mainly achieves the purpose of antioxidation by loosing two electrons and two protons to form L-dehydroascorbic acid [12]. Applying 10% ascorbic acid to the tooth surface after tooth bleaching is helpful to counteract the adverse effects of the adhesive bonding to the enamel [61], and ascorbic acid may also play the roles of polymerization promoter and co-initiator to have a positive effect on the adhesion of the composite resin to the tooth [62]. Garcia et al. [63] provided a one-year follow-up case report to further prove that immediate bonding after bleaching is trustable in dental clinics by using sodium ascorbate gel to avoid waiting time. However, a clinical trial by De Paula et al. [64] indicated that oral administration of ascorbic acid (500 mg, three times daily) was not a feasible approach to prevent bleaching-induced tooth sensitivity or reduce its intensity. Louzada et al. [65] investigated the anti-inflammatory potential of carvedilol gel in the pulp of rats after bleaching, where Carvedilol played the role of antioxidants. This histopathological study proved the effectiveness of Carvedilol in minimizing the damage of hydrogen peroxide, especially in deep resins of the dental pulp. Gupta et al. [66] assessed the antioxidant property of 10% amla extract on bond strength and color stability of power-bleached teeth, but, unfortunately, negative results were obtained. Vidhya et al. [18] reported another natural antioxidant of grape seed extract (oligomeric proanthocyanin complexes (OPCs)) which had neutralization effect on excessive free radicals of bleached tooth, and the effect was superior to ascorbic acid.The 5% OPCs and 10% ascorbic acid solutions had the ability to remove free radicals, and the effectiveness was 50 times that of 10% ascorbic acid [67]. As a natural plant metabolite, it has been proven safe in various clinical applications and dietary supplements as an antioxidant [68]. To improve the bioavailability of OPCs, Tian et al. [19] proposed a solid self-double-emulsified drug delivery system (SDEDDS) to deliver antioxidants. The double emulsification system is mainly composed of water-in-oil emulsion and hydrophilic surfactant, which can protect it from degradation and make it better absorbed by the small intestine [19,69].

### 3.2. Dental Implants

Periodontal disease leads to changes in the periodontal tissue, which in turn leads to the destruction of the alveolar bone. If it is not treated in time, tooth loss might be the consequence. Dental implantations are currently an effective method for the treatment of tooth loss [70]. Biomaterials used to make dental implants include metals, ceramics, carbons, polymers, and composites. Polymer materials are rarely used in implant dentistry and were only used to manufacture shock-absorbing assemblies placed between the implant and the superstructure [71]. As early as 1957, a Swedish surgeon studied bone healing and regeneration, and found that bone could grow together with titanium (Ti), and it could effectively be adhered to teeth without repulsion [72]. This also laid a foundation for the development of titanium dental implants. Since 1992, with the development of modern ceramics, ceramic surface treatments and ceramic-like elements have been incorporated into implants to further enhance osseointegration [73]. The titanium dental implants have attracted the most interest.

**Figure 3:**
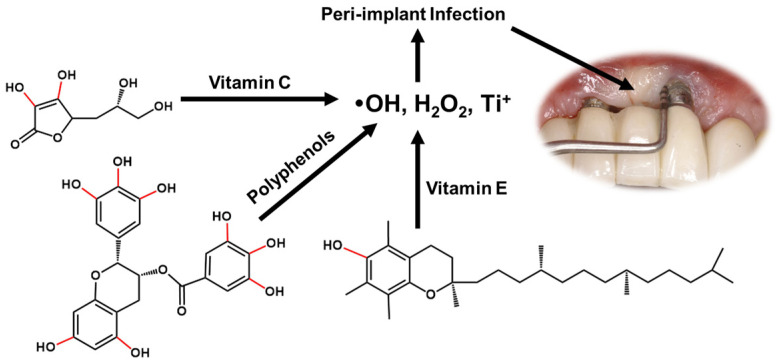
Antioxidants for the treatment of periodontitis during dental implants.

The process of dental implant implantation inevitably generates ROS. On one side, ROS are required for cell signaling and normal metabolism. On the other hand, excessive oxidative stress may lead to damage on DNA, ribonucleic acid (RNA), and proteins [74]. Tsarik et al. [75] reported that the oxide layer generated on the surface of titanium alloy implant may reduce the corrosion potential of the metal, while friction would lead to the rupture and corrosion of the titanium dioxide layer. From this point, the electrochemical reaction would occur. A large number of titanium ions were generated at the anode, and free radicals and hydrogen peroxide were generated as intermediate products. When titanium dioxide is corroded, hydrogen peroxide generated by electrochemical reaction will continue to react with titanium dioxide to form hydroxyl radicals [76]. Bressan et al. [77] analyzed the effects of titanium (Ti) particles on mesenchymal stem cells (MSCs) and fibroblasts (FU), and the 3-[4,5-dimethylthiazol-2-yl]-2,5 diphenyl tetrazolium bromide (MTT) test was used to evaluate cell proliferation and generation of ROS. It was found that titanium particles reduced the cell survival time and increased the generation of ROS. Accompanied by oxidative stress, bone regeneration imbalance was induced. The process of implant placement may lead Ti particles to enter the tissue and cause inflammation. Therefore, tetracycline, doxycycline, chlorhexidine, hydrogen peroxide, and citric acid can be used to remove excess Ti particles, among which hydrogen peroxide, citric acid, and chlorhexidine are more effective [78]. In addition, Abdulhameed et al. [79] reported that titanium dioxide nanoparticles (TiO_2_NPs) can induce oxidative stress, reduce osteogenesis, and damage the antioxidant defense system. After placement of a dental implant, friction and twisting can destroy the oxide layer on the surface, leading to an increase of ROS and an inflammatory, such as peri-implant (PI), infection may occur (Figure 3) [70,80]. Antioxidants such as ascorbic acid, polyphenols, and vitamin E are choices for treatment. PI and periodontal disease are manifested by soft tissue and bone damage, in which ROS play an important role in cell transmission, maintenance, and proliferation [81]. Dental implant materials are usually made of titanium or titanium alloys, and when exposed to oxygen, a layer of titanium oxide forms on the tooth surface to protect it [82]. In homeostatic conditions, ROS are involved in cellular maintenance, signal transduction, and repair of all tissues. However, if excessive ROS are accumulated, it can lead to oxidative stress, resulting in cellular damage and tissue destruction. Similarly, antioxidants protect immune cells by converting free radicals into waste products affected by the destructive effects of ROS.

Typically, a certain amount of ROS is already present before the implant is implanted [83]. Antioxidants have a protective effect on periodontal tissue and can neutralize ROS to prevent tissue damage [84]. At present, antioxidants in the treatment of local inflammatory reactions, such as periodontitis, usually quickly disappear with reactive oxygen species and other free radicals. The lower treatment effect might correspond to the antioxidants delivery. When the inflammation is serious, there are redness, swelling, bleeding, wound split, and other conditions. Most of these antioxidants can be obtained from diets and supplements, and supplements can be taken in addition to the diets mentioned above [2]. Tannic acids are macromolecules consisting of a central glucose molecule linked to 10 surrounding gallic acid units [85]. Thus, tannic acids have a large number of functional groups but are still water-soluble and hydrolysable [86,87]. Huang et al. [88] modified the Ti implant surface with Ag nanoparticles incorporating tannic acid/nanoapatite composite coatings, and their antibacterial and antioxidative properties were highlighted. The slow release of tannic acid in this study is favorable to the persistent antioxidative activity of the dental implant. Maruyama et al. [89] investigated the effects of dentifrice containing green tea catechins using a rat model, and up to 8 weeks of follow-up proved the efficacy of green tea catechins to prevent periodontal inflammation by decreasing gingival oxidative stress. Quercetin is a natural flavonoid [9], and is an antioxidant that has both anti-inflammatory and antioxidant properties [90,91]. Catauro et al. [92] entrapped quercetin in a silica/poly(ε-caprolactone)-based hybrid material by a sol–gel route to use as novel dental implant, and the hydrogen-bonded reactions between quercetin, silica, and polymer matrices were considered as the key to produce antiradical efficacy. Polyphenols are natural compounds with antioxidant and antimicrobial properties and aromatic benzene rings containing multiple hydroxyl groups [93,94]. The antioxidant activity of polyphenols scavenges free radicals by supplying hydrogen atoms from hydroxyl groups in the phenolic ring [94]. Additionally, polyphenols exert their antioxidant function through their ability to chelate iron and other metal ions, thereby preventing the catalytic oxidation of hydrogen peroxide and superoxide to hydroxyl radicals [95,96]. Polyphenolic compound curcumin can also lead to an increase in the level of the antioxidant enzyme glutathione peroxidase, which reduces the ROS level in cells [97]. Vitamin E is a common antioxidant with the highest concentration in human mitochondria. The main mechanism of action of vitamin E is to interact with superoxide in mitochondria, limit its formation, stabilize mitochondrial membrane, and remove antioxidants that have been generated [98]. Vitamin E could be added to implant formulas for antioxidative purposes, and a clinical study [99] indicated that adding low concentrations of vitamin E (less than 0.1%) did not affect the physical and mechanical properties and can prevent oxidation for up to 24 months post-implantation.

At present, antioxidants in the treatment of local inflammatory reactions, such as periodontitis, usually quickly disappear with ROS and other free radicals. The lower treatment effect might correspond to the antioxidants delivery. Ozawa et al. [100] reported a new material in which hydrogels containing nitrogen oxide radicals were injected into experimental rat models with PI and in vitro osteoblasts. These hydrogels can reduce the presence of ROS, inhibit hydrogen peroxide and lipid peroxidation, and can increase the retention time of antioxidants during treatment. After injection, the gel was assembled by nanoassembly, and the micelles containing nitroxide radicals were partially decomposed [13]. These redox nanoparticles usually remove extracellular ROS without affecting the normal cellular reactions. Therefore, the delivery of antioxidants by gel encapsulation is worth studying for the treatment of oxidative stress conditions such as periodontal diseases caused by dental implants.

### 3.3. Dental Restorations

Dental filling materials have been widely used as a means of dental restoration in recent years, mainly aimed at repairing teeth damaged by dental caries or trauma. There are three main types of filling materials, which are amalgam, dental resin composites, and glass ionomer cement (GIC) [101].

#### 3.3.1. Dental Amalgam

Dental amalgam is one of the most widely used restorative materials in dentistry. Despite the continuous progress in the development of dental fillings, amalgam is still widely used due to its durability, low price, easy use, and other advantages [102]. However, there are still many disputes about the use of amalgam: the continuous release of mercury vapor in dental amalgam, the possible formation of organic mercury in the mouth, the impact of mercury exposure on human cell gene regulation, and the relationship between amalgam and Alzheimer’s disease, Parkinson’s disease, and other diseases [103]. The alloys currently used are 40 to 70% silver, 12 to 30% tin, and 12 to 24% copper. It may also include 0–4% indium, 0.5% palladium, and more than 1% zinc. Zinc prevents the oxidation of other metals in the alloy during manufacturing. The powder alloy is mixed with liquid mercury, then soft amalgam putty is poured into the alveolar bone to cure. Cured amalgam fillings are cheaper, stronger, and last longer than other types [103].

Mercury penetrates into the brain through the oral area and accumulates in the brain, thereby affecting the central nervous system [103]. Mercury in amalgams is responsible for the redox imbalance in the system. Mercury in amalgams usually exists in various oxidation states (Hg^+^ and Hg^2+^) and is prone to react with cysteine and glutathione to form sulfide, which is methylated by bacteria to form highly toxic methylmercury (MeHg) or dimethyl mercury (Me_2_Hg) and organic compounds [104]. The catalytic oxidation of mercury has also been the focus of attention. Similar to other metal ions, mercury interacts with most sulfhydryl (-SH) groups and produces living ROS, such as superoxide anion, hydrogen peroxide, and hydroxyl radical, thus inducing oxidative damage in tissues. Pizzichini et al. [105] conducted a clinical study towards the release of mercury from dental amalgam, and they found a significant negative correlation between total antioxidant activity (TAA) and mercury levels in females but not males. Another clinical study, by Lindh et al. [106], suggested that the metal exposure from dental amalgam did cause ill health, and the removal of dental amalgam coupled with antioxidant therapy were supportive to improve the quality of life in patients.

Amalgam restorative systems currently in use typically include encapsulated and predosed presentations, which are applied with isolation of the tooth by means of a rubber dam both for insertion and removal. Couple with the high-powered suction during operation, the possible toxic effect of amalgam is actually very limited. As for the potential mercury release and induced oxidative stress, an appropriate application of antioxidants is necessary. Cysteine, homocysteine, and GSH play the role of antioxidants in the body, and GSH maintains the oxidative balance of cells by quenching free radicals. As an important defense against oxidative stress, GSH increases the antioxidant capacity of mitochondria and protects mitochondria by resisting lipid peroxides produced by hydrogen peroxide, hydroxyl radicals, and mercury [107]. Due to the poor selectivity of GSH, it may not only remove toxic ions, but also eliminate the ions necessary for the human body.

**Figure 4:**
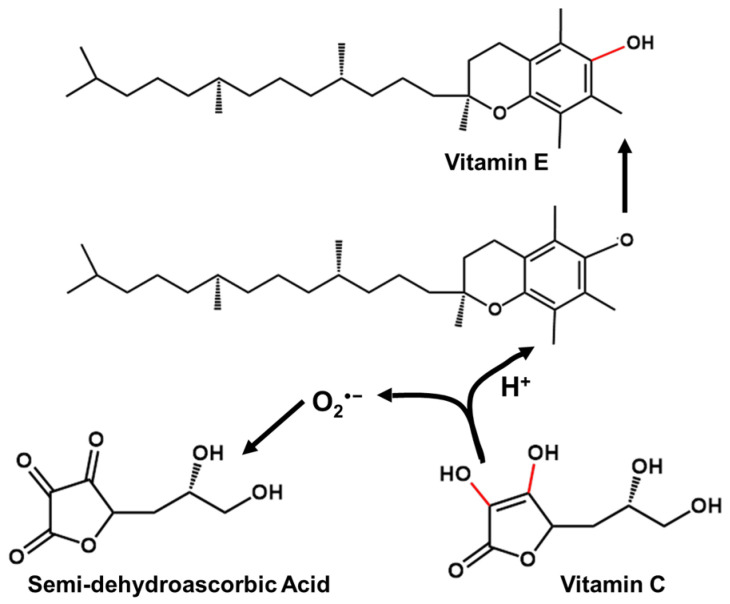
Antioxidative mechanism of vitamin C.

Antioxidants such as vitamin C, vitamin B complex, vitamin E, and niacin are usually taken to supplement the treatment [106]. Fisk et al. [108] investigated the correlation of dental amalgam restorations supported by antioxidant therapy (vitamin B complex, vitamin C, vitamin E, and sodium selenite), and the systemic route was proved to be effective. Vitamin C as an antioxidant is discussed here. The antioxidant effect of vitamin C is manifested in the rapid reaction with O_2_^−^, HOO·, and OH to produce semi-dehydroascorbic acid, and can also restore the prototype of oxidized vitamin E [109] (Figure 4). Because a large amount of vitamin C may cause damage to the stomach and intestines, excess vitamin C is excreted with body fluids. Vitamin C has the advantages of antioxidation and antiaging, but the sustainability of vitamin C in light, oxygen, and heat is very low. In order to extend the shelf life of vitamin C, encapsulation technology is used to wrap it. Common packaging materials include liposomes, nanoparticles, and microcapsules [110]. Additionally, according to Gallusi et al. [111], dental amalgam was not as toxic as expected, and human clinical studies have shown no increased risk for systemic diseases or conditions compared with composite restorations. Therefore, a systemic route is applicable for the delivery of antioxidants. 

In practical clinical applications, the uses of dental amalgams are controversial at present. The Norwegian Ministry of Environment banned the use of amalgam on January 2008, but the Scientific Committee of European Commission did not follow this ban because all available direct restorative materials have drawbacks and potential biological side effects. Alkhudhairy [112] investigated the attitudes of dental practitioners working in Riyadh, Saudi Arabia, towards the use of dental amalgam, and the results were strongly gender-dependent. Male participants did not oppose the use of amalgam in their clinical practices, and did not consider it an occupational risk factor. However, female participants held significantly different options to those of males. Joshi et al. [113] limited their studies to males, and investigated the mercury level difference between dentists and nondental health professionals. No relationship was found between these two groups, and consumption of saltwater fish was considered as the primary exposure factor. A clinical report by Broadbent et al. [114] demonstrated that the decreased use of dental amalgam has been a global trend despite having no official policy. The tooth-colored direct restorations, particularly composites, have become the most frequently used filling materials in dental clinics.

#### 3.3.2. Dental Resin Composites

In the last century, composite resin was introduced into dental repair as a filling material, such as for caries, and tooth structure damaged by erosion or fracture [115]. Resin composites are also widely used because of their good compressive strength and aesthetic properties. Dental resin composites are usually composed of two parts, a small part of polymerizable free radicals and a large part of inorganic fillers, which mainly include quartz, ceramics, and silica [116]. Commonly used monomers are 2-hydroxyethyl methacrylate (HEMA), bisphenol A-glycidyl methacrylate (Bis-GMA), and co-monomer triethylene glycol dimethacrylate (TEGDMA) [117]. These fillers and monomers are mixed in a certain proportion, and a certain initiator is added. After heat or light irradiation, they can be solidified. Finally, after grinding and polishing, the tooth repair is completed. However, the composite resin has potential toxicity in the process of polymerization and release, and some added compounds, such as monomers and initiators, may produce a certain amount of free radicals, and even lead to oxidative stress [118].

**Figure 5:**
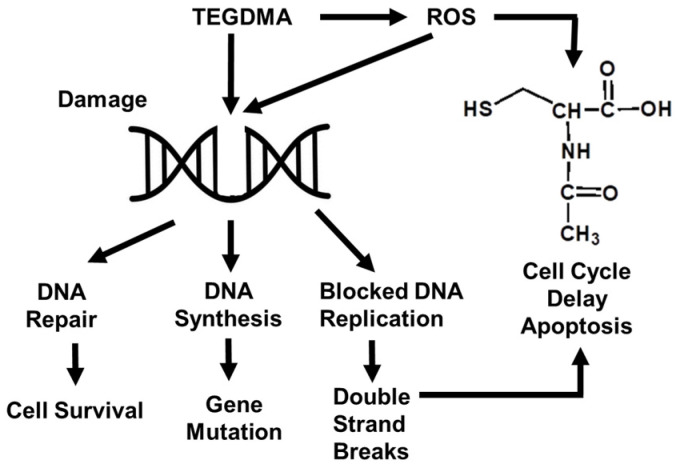
Damage to cell function by monomer HEMA released by resin restorations.

The polymerization process of composite resin is typically incomplete. The monomer HEMA released from the resin restorations may interact with living tissues in the oral environment, producing cytotoxicity, and the immune cell response and the function of odontoblast cells may be affected (Figure 5). It was also reported that apoptosis of odontoblast-like cells, undifferentiated pulp cells, or macrophages in mice repaired with composite resins depended on the degree of adhesion resin polymerization [119,120]. In addition, a low degree of conversion causes the unpolymerized monomers to be released into the oral environment, which may reduce the mechanical properties of the composite and accelerate the degradation. The generation of free radicals and ROS when applying dental composite materials is mainly related to the induction of monomers in the composite materials [119]. Methacrylate in dental resin may lead to the consumption of GSH, but NAC generated by GSH may alleviate this situation. Schweikl et al. [119] demonstrated that resin monomer damages mitochondria, consumes the antioxidant glutathione in cells, reduces its free radical scavenging ability, increases the production of ROS, and accelerates oxidative damage. Among them, epoxy resins and acrylic monomers are listed as important occupational sensitizers and have the potential of cross-reaction [119]. By 2021, the European Food Safety Authority (EFSA) had reduced the tolerable daily intake of bisphenol A to 0.04 ng/kg. If the amount of exposure is too high, it may lead to tooth sensitivity in clinic and even affect the health of the body [121]. When concentrations reach a certain amount, the composition of the composite may alter cytokine secretion in human monocytes. Recent studies also found that camphorquinone (CQ), as an initiator of composite resins, is also considered to be a compound that produces ROS. In addition, the use of ultraviolet curing in composite resin restoration is also an aspect in the production of ROS.

Antioxidants have been considered when using dental resin composites. It has been proven that antioxidant-added binders produce less ROS and are correspondingly less toxic [122]. Antioxidant polymers with molecules of natural antioxidants (e.g., quercetin and curcumin) have also been synthesized for attenuating material-induced oxidative stress [123]. GSH and NAC are antioxidants responsible for the reduction of DNA damage caused by CQ oxidation at relatively higher concentrations. The addition of NAC in resin-based materials favors the formulation of a new material in which the intrinsic cytotoxicity of the resin was potentially detoxified [124]. Flavonoids are powerful antioxidants that can scavenge free radicals in the body and have antibacterial and disinfectant properties.

Vitamin E is a common antioxidant, with the highest concentration in human mitochondria. Vitamin E also has the function that other antioxidants do not have, namely, regulating the activity of related enzymes. It can inhibit the activity of protein kinase C and inhibit the production of monocytes [125]. The absorption of vitamin E is mainly in the small intestine. Since direct oral administration may destroy the strong acid in the stomach and lose its activity, a functional ingredient delivery system is needed to retain its activity and improve stability [14]. Zhang et al. [15] mentioned a new oral delivery-responsive intelligent hydrogel in their research a few years ago. It forms a spatial network structure through macromolecules and can change its performance through environmental conditions or interface reactions [126]. Carboxymethyl starch is used as the carrier material, and the carboxyl group on the starch macromolecule is related to the hydration, swelling, and solubilization of the delivery carrier [14]. Another material of the hydrogel is xanthan gum, which is acid-resistant and antienzymatic. Research has shown that xanthan gum is helpful to delay the release, making vitamin E stay longer [127]. The microcapsules containing vitamin E were prepared by spray-drying the mixture of carboxymethyl starch and xanthan gum, and are able to reach the upper part of the small intestine and improve the bioavailability and efficiency of vitamin E [14].

#### 3.3.3. Glass-Ionomer Cement (GIC)

GIC is a dental translucent cement, which is the product of the reaction of ion-leachable glass and polyacrylic acid aqueous solution [128]. In terms of performance, although it is not as perfect as resin composite material, it has a wider clinical application because it can control the performance according to changing the ratio [129]. The advantages of GIC are strong adhesion with teeth, low risk of tooth corrosion, and more realistic and beautiful color [130]. GICs are widely used in artificial crowns, orthodontic brackets, and cavity liner [131]. In order to further improve the mechanical strength, resin-modified GIC (RMGIC) was developed, and can be photocured by adding monomers [132]. The monomers used here are typically bis-GMA and HEMA [133]. The monomers added in RMGICs are the main source of free radicals, which may cause certain oxidative damages. When resin components, such as monomers and initiators, are added to the traditional GIC formula, the residual HEMA monomer easily spreads through the dentin tubules to the pulp cells, stimulating the production of free radicals. Meanwhile, the antibacterial substance fluoride added to the GIC will also bring certain cytotoxicity, which may cause pulp damage [134]. The clinical manifestations are pain when the teeth are irritated by heat and cold, or bleeding gums. Generally, vitamin E is used as an antioxidant, and its mechanisms of action and delivery are similar to those of the dental resin composites.

## 4. Conclusions and Perspective

As people are paying more and more attention to oral health, the details about dental procedures are also growing. This has greatly improved people’s oral conditions and promotes the innovation and progress of dental materials. This article focused on the applications of antioxidants in dental procedures. For each dental procedure, the types of antioxidants used in dental treatment, the mechanism of action, and delivery of various antioxidants were reviewed. The challenges and safety assessment of these materials in the current field were also discussed.

In various dental procedures, antioxidants are highlighted for their significance in the process of scavenging free radicals and repairing damaged cells. However, determining how to select the most appropriate antioxidant according to a certain type of free radicals generated in dental procedures, how to deliver these antioxidants to maximize their effectiveness, and how to balance their toxicity and bioavailability still need further studies. Additionally, scientific trials that support the widespread use of various antioxidants in dental clinics are of limited validity. Future works focused on antioxidants delivery and bioavailability assessment are highly recommended. It is also urgently necessary to conduct clinical studies, especially the long follow-up period studies in dental clinics, to further confirm the appropriate antioxidative approach for human usage. The explorations of advanced applications of antioxidants in the dental field are still underway.

## Figures and Tables

**Table 1:** Mechanism of action and delivery of some exogenous antioxidants.

Antioxidants	Mechanism of Action	Selected Delivery Form	Ref.
Vitamin C	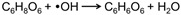	Gel assembled from nanoparticles.	[12,13]
Vitamin E	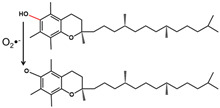	Responsive intelligent hydrogel.	[14,15]
Quercetin	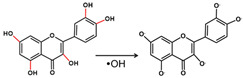	Quercetin-loaded nanoemulsion (QNE), polymeric nanoparticles, liposomes.	[16,17]
Oligomeric proanthocyanin complexes (OPCs)	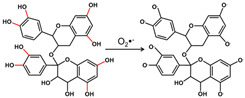	Solid, self-double emulsified drug delivery system (SDEDDS).	[18,19]
